# Mr. R. N. Starr, in Reply to Dr. Kinglake, on the Obstetric Practice

**Published:** 1816-05

**Authors:** 


*For the London Medical and Physical Journal.*

### Mr. R. N. Starr, *in Reply to* Dr. Kinglake, *on the Obstetric Practice.*

In your Journal for this month, page 174, I was struck with the further remarks of Dr. Kinglake on the Obstetric Practice, which he so strongly opposes should be under the management of male practitioners, excepting but in preternatural cases, where he modestly allows a medical man may be consulted.

I am afraid the Doctor, when speaking of the too frequent interference of the accoucheur in cases of parturition, (which, I consider, is rather exaggerated,) did not remark the practice of the female midwife, who, I think he will find, are much more officious in the application of the *Tartus Eruditus* than the male practitioner. Dr. K. seems prejudiced against the practice of man-midwifery. I am afraid he has seen little of the practice of the female midwives, or probably he would not have formed those illiberal ideas with respect to the former. I have a better opinion of my medical brethren than to think few regular practitioners labour under the charge imputed against them by the Doctor. With regard to the female African and uncivilized American, are they not in a state of nature? and we are all aware, that the increase of luxury debilitates the human constitution. If we look at the brute creation, we shall find, as they become domesticated, the females consequently suffer more during parturition, than when in a state of nature. This, I consider, is in consequence of artificial feeding, and confinement. We shall find that the females in tropical climates suffer less during during the parturient state than those in more northern regions: this may probably happen from a greater laxity of habit, together with abstemious living, for we shall find that animal food is little used amongst them, or even any other luxury. With due deference to the professional abilities of Dr. K., I should recommend him to study the old proverb—*Sit verbum sapienti.*

*Barnsley;*

